# Syrian-born children with a refugee background in Rotterdam. A child-centred approach to explore their social contacts and the experienced social climate in the Netherlands.

**DOI:** 10.1080/17482631.2020.1721985

**Published:** 2020-12-09

**Authors:** Barbara van der Ent, Jaco Dagevos, Talitha Stam

**Affiliations:** Department of Public Administration and Sociology, Erasmus University Rotterdam, Rotterdam, The Netherlands

**Keywords:** Children with a refugee background, peer relations, inclusion and exclusion, symbolic boundaries, social boundaries, boundary work, child-centred approach, focus groups, Syria

## Abstract

**Purpose**This article explores how newly-arrived children with a refugee background describe their everyday lives in the Netherlands, with a focus on how they perceive their peer relations and the broader social climate in the host country. **Methods**In this case study, focus groups were conducted with 46 Syrian-born children with a refugee background, ranging between the ages of 8 to 17 years old. All participants have a temporary residence permit and live in Rotterdam together with (part of) their family. A board game was developed as a research tool to stimulate children to share their perspectives on their friends and experiences with inclusion and exclusion. **Results**An important finding is that all of the children have friends in the Netherlands. The majority of their friends have an Arab background, and different reasons for this composition are discussed. Furthermore, although all of the children expressed that they feel welcome in Dutch society, they had also encountered exclusion, which generates emotional responses. **Conclusion**Using a theoretical boundary perspective, we show that children are involuntarily subjected to symbolic boundary drawing by others, while taking part in boundary work themselves too. Within the domains of the children’s social networks and the broader social climate in the Netherlands, we further examined the relations between symbolic and social boundaries.

## Introduction

Imagine that you were born in Damascus, Syria, in the 2000s. You went to school, spent a lot of time with your family, played with your friends on the streets, had dreams, and made plans for the future. But then, war broke out and many things changed. After a while, your parents decided that it was necessary for you and your family to leave Syria and flee to Europe. You arrived in the Netherlands, perhaps after a series of temporary stops in other counties. After spending some time in an asylum seekers’ centre, your family was assigned to the city of Rotterdam. You are happy that you can go to school again, even though it is difficult not being able to speak or understand Dutch yet. You live with part of your family, but you miss other and extended family members. You are once again able to play in the streets, but first you need to make new friends to play with. You have the impression that some people are not happy with your presence in this country, and you have to adjust your dreams and plans for the future to your new country and new situation.

This paper is about newly-arrived children with a refugee background^1^ in Rotterdam, the second largest city of the Netherlands. This study explores the case of Syrian-born children. In recent years, Syrians have become the largest group of refugees in the Netherlands and almost 40% is aged 0 to 18 years (CBS, 2018). Most of them live together with their families. In Rotterdam, there are 700 Syrian-born children under the age of 18, who make up 40% of the Syrian population with a refugee background in that city (Basis Registratie Personen, 2018). Within the highly diverse context of Rotterdam—more than 50% of the population has a migration background and the city counts more than 170 nationalities (Gemeente Rotterdam, 2018)—this study shows how newly-arrived children try to make sense of the global society in which they live.

People with a refugee background, and in recent years more specifically Syrians, are the focus of several research projects. Studies focus on adult refugees (Dagevos, Huijnk, Maliepaard, & Miltenburg, 2018; Heelsum, 2017; Roblain, Malki, Azzi, & Licata, 2017), approach children’s situations from the viewpoint of adults (Miltenburg & Huijnk, 2018), or ask adults retrospectively about their youth as a child refugee (Luster, Qin, Bates, Rana, & Lee, 2010). When children’s perspectives are taken into account, the focus is often on unaccompanied minors (Bean, Eurelings-Bontekoe, & Spinhoven, 2007; Groark, Sclare, & Raval, 2011; Luster et al., 2010; Wallin & Ahlström, 2005), as this group is perceived as being particularly vulnerable.

In this research, the focus is on children with a refugee background who live together with their family. We use a child-centred approach, which means we focus on children’s perception of their lived experiences (Ensor & Gozdziak, 2010; Gardner, 2012). In this explorative study, focus groups with 46 Syrian-born children (aged 8 to 17 years) were conducted to give these children the opportunity to share experiences they see as relevant. Their stories steered the direction of the conversations to a substantial extent.

**Figure 1.**
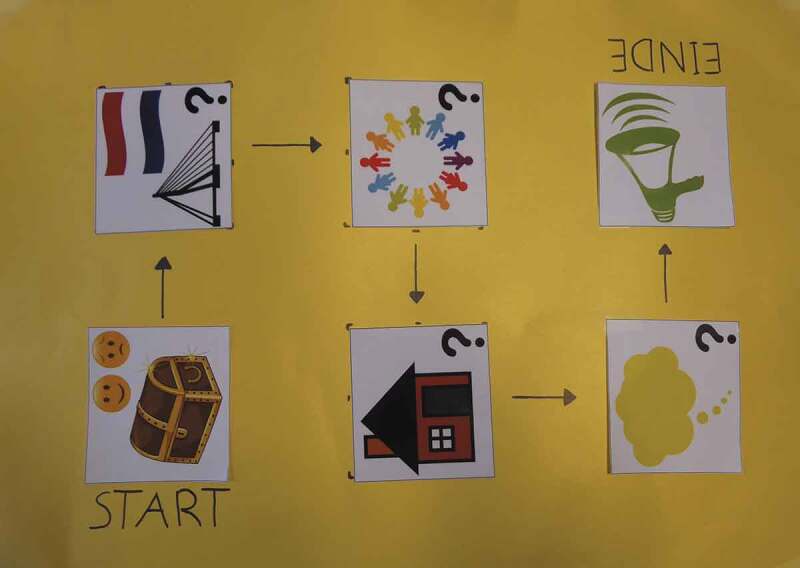
The board game that was developed as a research tool for this study

**Figure 2.**
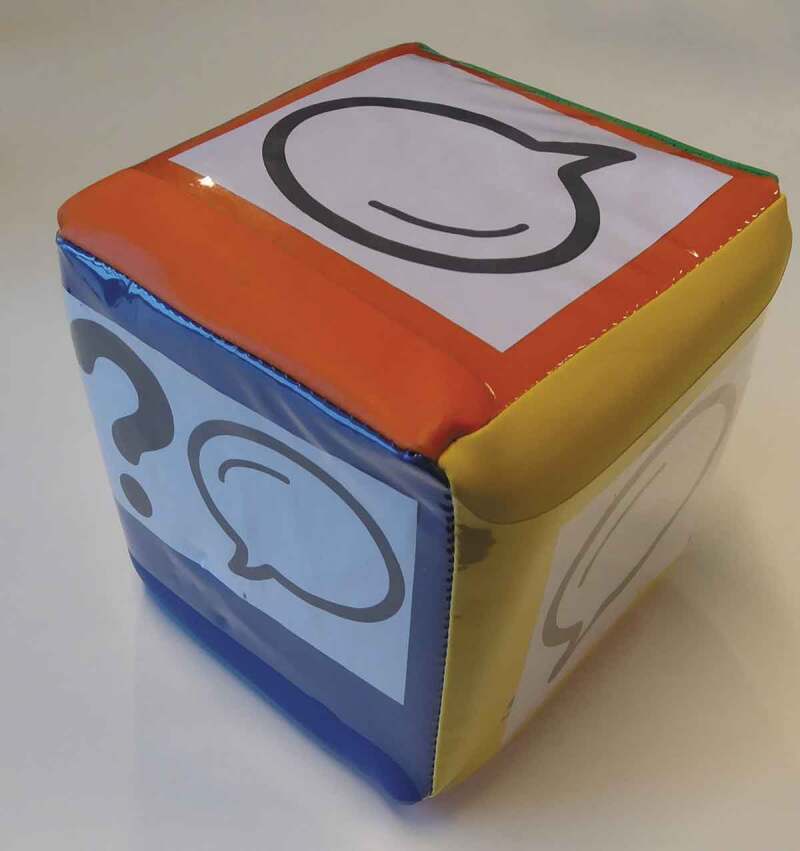
The dice that was used to determine if children had to answer a question or if they could also read it aloud

We focus on children’s lives after their flight, when they and their families have received (temporary) residence status, and are facing up to resettling and integrating in a new country. In the post-flight phase, refugees must deal with large social, cultural and linguistic differences between the place of origin and the new setting (Fazel, Reed, Panter-Brick, & Stein, 2012). The living circumstances in the post-flight period are highly important to establishing a child’s sense of well-being, resilience and ability to deal with their new situation (Van Schie & Van den Muijsenbergh, 2017). Although adversities from the past can still have an influence, Almqvist and Broberg (1999) stress that “[f]or many refugee children, however, current life circumstances in receiving host societies, such as peer relationships and exposure to bullying, are of equal or greater importance than previous exposure to organized violence” (Almqvist & Broberg, 1999, p. 729).

This paper explores how children with a refugee background experience living in the Netherlands. More specifically, it focusses on two social domains: children’s networks of peers and how they perceive the broader social environment of the Netherlands, including their feeling of being welcome and their experiences of social exclusion. The research questions are related to these domains: 1) how do these children evaluate their social contacts with peers, and 2) what are their experiences within the broader social environment in the Netherlands? These two social domains were chosen for multiple reasons. First, during our research the children elaborated extensively on these topics, indicating that they are relevant aspects of their lives. Second, children’s social networks and the broader social environment are important factors that can affect their well-being (Correa-Velez, Gifford, & Barnett, 2010), mental health (Bean et al., 2007; Fazel et al., 2012; Lustig et al., 2004; Van Schie & Van den Muijsenbergh, 2017), resilience (Sleijpen, Boeije, Kleber, & Mooren, 2016), and subsequently their integration in the host society. In other words, these social domains affect the extent of the predicament of children with a refugee background.

This study contributes to a deeper understanding of day-to-day life experiences of newly-arrived children and youth, who have resettled with their families in an ethnically diverse European city. It shows how young individuals with a refugee background make sense of their lives in a new society. Theoretically, this article builds further on the boundary perspective (Alba, 2005; Lamont & Molnár, 2002), which regards boundaries between groups as dynamic and constructed, and the result of boundary work. Boundary concepts are useful in the exploration of children’s perspectives on group thinking, which is relevant for both domains. The article contributes an analysis of how young individuals are involuntarily subjected to boundary drawing by others, while also undertaking boundary work themselves.

## Theoretical framework

Previous studies that focus on children with a refugee background have tended to use theories from a health perspective and elaborate on risk and protective factors that affect children’s psychosocial situation or mental health. These studies mainly used quantitative measures, including pre-flight, flight and post-flight indicators (Almqvist & Broberg, 1999; Bean et al., 2007; Correa-Velez et al., 2010; Kia-Keating & Ellis, 2007), or conducted systematic literature reviews to gain insights into important stressors and chances for successful social adjustment (Fazel et al., 2012; Lustig et al., 2004; Sleijpen et al., 2016; Van Schie & Van den Muijsenbergh, 2017). Another strand of research is directed towards the educational situation of newly-arrived children and focuses, for example, on their aspirations (Shakya et al., 2012), opportunities and obstacles in their school trajectories (Crul, Keskiner, Schneider, Lelie, & Ghaeminia, 2016; McBrien, 2005). Research that took a qualitative approach and included children themselves (often in combination with consulting adults or experts) primarily used individual semi-structured interviews to investigate experiences with peers (Anderson, 2001; Beirens, Hughes, Hek, & Spicer, 2007), well-being (McFarlane, Kaplan, & Lawrence, 2011) or focused on unaccompanied minors (Groark et al., 2011; Luster et al., 2010; Wallin & Ahlström, 2005). Studies exploring the experiences of newly-arrived children using focus groups have been conducted by Earnest, Mansi, Bayati, Earnest, and Thompson (2015) in Australia and by Selimos (2017) in Canada. Both studies broadly explored youth perspectives on matters such as their new country, social support and sense of belonging, and underline the importance of inclusion and positive peer relations. Earnest et al. (2015) used a psychosocial framework with a focus on well-being, whereas Selimos (2017) theorized the nexus of different social status positions of youth with a refugee background. Both studies based their findings on a small number of focus groups (n = 3) with a population of 16 years and older, from different origins, some of whom were unaccompanied minors. In our study, we focus exclusively on the perspectives of Syrian-born children who are mostly younger and living with their families.

In this study, a theoretical boundary perspective will be used, since this can contribute to an understanding of group thinking that is relevant with respect to children’s experiences with social contacts and the broader social environment. In this perspective, intergroup relations and the boundaries between groups are seen as being dynamic and interactive. Ethnic groups do not exist “out there”, but are rather produced in social interaction. Boundaries between groups are always constructed (Witte, 2017), and can—depending on social circumstances—arise and disappear, or be crossed, shifted or blurred (Alba, 2005).

Lamont and Molnár (2002) make a distinction between social and symbolic boundaries. Symbolic boundaries show a categorical distinction. They divide people into groups, such as Dutch or foreigner, black or white, Muslim or Christian. These boundaries create feelings of solidarity; they show who is in your in-group and who is not. Social boundaries, on the other hand, are concerned with distinctions in behaviour; because someone belongs to another group (symbolic boundary), this person is treated differently (social boundary). Thus, although social and symbolic boundaries can be separated conceptually, they are almost always intertwined in practice. They arise when “actors distinguish between different ethnic categories and when they treat members of such categories differently” (Wimmer, 2013, p. 3). For example, when thinking about a newly-arrived child, a symbolic boundary is drawn between these children and children who are native to Rotterdam. A corresponding social boundary could imply that the newly-arrived child is excluded from playing a game and a Rotterdam-born child is not, or that a child with a refugee background receives more attention and help at school because he or she is a refugee. This study will highlight how Syrian-born children are involuntarily made the subject of symbolic boundaries, but also how they themselves draw boundaries between different groups. This is an important characteristic of this boundary approach; everybody is involved in boundary work (Witte, 2017).

The boundary approach is useful in the exploration of children’s perspectives on their social network, since symbolic boundaries can influence who your friends are. Social contact with peers is important for children with a refugee background, as it is for all children. Positive peer relationships—in other words, friends—promote emotional well-being and feelings of self-worth, as well as fostering resilience (Almqvist & Broberg, 1999; Van Schie & Van den Muijsenbergh, 2017). Children who have a good social network and peer support are less likely to develop mental health problems; rather, they have greater feelings of safety and protection (Beirens et al., 2007; Van Schie & Van den Muijsenbergh, 2017). Feeling included and valued by others will help them to feel worthwhile and competent (Suárez-Orozco, 2004). Furthermore, having friends to play with contributes to their social adjustment to the host society (Suárez-Orozco & Todorova, 2003). Unfortunately, making new friends and building a supportive social network can be difficult, because newly-arrived children can encounter discrimination and bullying (Fazel et al., 2012; Kia-Keating & Ellis, 2007). Almqvist and Broberg (1999) showed that Iranian children in Sweden reported far more instances of bullying than other children. Anderson (2001) states that in Germany, “refugee children have had experiences of being made to feel outsiders by other children” and feel excluded when other children say “You little foreigner, you don’t belong here […] we don’t need people like you here” (Anderson, 2001, pp. 190–191). This quotation clearly illustrates how symbolic boundaries are drawn—with words such as *outsiders* and *foreigners—*and are linked to social boundaries of bullying and exclusion.

Furthermore, the boundary concept provides insights into the exploration of the social climate in the broader society and children’s experiences of inclusion and exclusion. For children with a refugee background, “the broader social environment within which they live their lives is crucial for positive reinforcement of being socially valued, of belonging, and of being able to participate in and contribute to society” (Correa-Velez et al., 2010, p. 1406). Similarly, Suárez-Orozco and Suárez-Orozco state that a significant factor in shaping children’s experience is “the general social and cultural climate they encounter. This climate is largely shaped by the general attitudes and beliefs held by members of the new society about immigration and immigrants” (Suárez-Orozco & Suárez-Orozco, 2001, p. 36), or in this case, beliefs about refugees. Thus, being included—a social boundary—plays a critical role in how children fare in their new country. Unfortunately, earlier studies show that people with a refugee background can be confronted with suspicion and fear (Beirens et al., 2007; Fantino & Colak, 2001; Suárez-Orozco, 2004), and may experience discrimination and racism. Perceived discrimination, experiences of social exclusion (such as bullying), and subjective social status are highly important predictors of newly-arrived youth’s sense of well-being (Correa-Velez et al., 2010; Fazel et al., 2012; Van Schie & Van den Muijsenbergh, 2017). In terms of boundaries, social boundaries of inclusionary and exclusionary behaviour may exist within the broader social climate and are related to the symbolic boundaries that are drawn. Based on earlier research on the social inclusion of children with a refugee background (Almqvist & Broberg, 1999; Anderson, 2001; Spicer, 2008), anti-Muslim sentiments (Kassaye, Ashur, & Heelsum, 2016; Maliepaard, 2012), and the divided social climate regarding refugees in the Netherlands (Van der Velden, 2015), it can be expected that Syrian children in the Netherlands encounter some forms of social exclusion related to different symbolic boundaries.

## Research methods

This study is based on the analysis of ten focus groups and one interview^2^ with Syrian-born children, conducted in the summer and fall of 2018. Focus groups provide insights into processes of collective meaning-making, because participants interact with each other. Additionally, focus groups give participants the opportunity to bring up topics they consider to be relevant (Bryman, 2008).

A total of 46 children (21 girls and 25 boys), aged 8 to 17 years old, participated in the focus groups. All of the children had come to the Netherlands from Syria as a refugee, had a temporary residence permit and were living together with at least part of their family in different neighbourhoods in Rotterdam. They had been living in the Netherlands from between 9 months to 4.5 years. The focus groups were mainly conducted in Dutch, but Arabic was spoken as well. Almost all of the focus groups contained children of a similar age and in most cases they knew each other beforehand. This fostered their trust and willingness to share their thoughts. Four focus groups consisted of only boys, two consisted of only girls, and the rest were mixed. This gender composition was partly due to the children’s availabilities and presence but was deliberately planned in the case of three same-sex focus groups with older children (15+ years old) to increase their openness to talk.^3^ The focus groups lasted between 1.5 and 2 hours.

Most children (n = 36) in this study follow the private integration programme run by *Stichting Nieuw Thuis Rotterdam* (SNTR, in English: Foundation New Home Rotterdam), an organization financed by the philanthropic foundation *Stichting De Verre Bergen*, which aims to facilitate the integration of a group of Syrian families in Rotterdam. This specific study of children is part of a larger research project (*EUR Bridge Research Project*) that studies and evaluates the SNTR programme and the integration of its participating families. Ten children were from families that are not participating in the SNTR programme but are receiving regular integration services from the municipality. Children were selected in multiple ways, depending on the location of the focus group and the information we had access to.^4^

Elaborating upon research ethics is especially important in research with a potentially vulnerable population and an expected power imbalance between researcher and participants. We will describe three topics—informed consent, research setting and the research tool used—to illustrate our ethical considerations.^5^

Parents and children were well informed about the purpose of this study so that they could make an informed decision regarding participation. Parents of children under the age of 16 were informed about the study with an explanatory letter, written in Dutch and Arabic, and asked for active consent. Children received an oral explanation and an informed consent form with text and pictograms (Danby & Farrell, 2005), which they all decided to sign. During the focus group, we checked if ongoing consent was still given by taking children’s participation and reactions into account (MacNaughton & Smith, 2005).

Focus groups were conducted at different locations: a summer school for Syrian children organized by SNTR (4x); language schools attended by the children’s parents (4x); the premises of a refugee organization (1x) and a secondary school offering transition classes (2x). In all cases, the children were “insiders” to these locations, which helped to make them feel comfortable and in control and reduced the power imbalance between participants and researcher (Horner, 2000; Punch, 2002).

From an ethical point of view and to increase the validity of the study, the research methods we used needed to be appropriate for the participants. In this case, it was important to make a connection with the children’s life world, give them control over what was happening, and relate to their capacities (Fargas-Malet, McSherry, Larkin, & Robinson, 2010; Punch, 2002). For this reason a board game (figure 1) was developed by the first author as a research tool (Crivello, Camfield, & Woodhead, 2009; Greig, Taylor, & MacKay, 2013). This game consisted of pictograms that corresponded with the themes of the discussion. Because the researchers chose the themes that were discussed, the children were probably made more aware of these topics than they usually are. In this article, we discuss two of these themes: “friends” and “living in Rotterdam and the Netherlands”.^6^

To actively involve the children in the focus group, the researchers asked them to choose a coloured pawn to play with. The children then decided the sequence of the themes as a group. Questions were written down on question cards and children rolled a large, coloured dice (figure 2) to determine if they only had to answer the question or if they could also read it aloud. Reading the questions from the cards turned out to be very popular, even for children with limited Dutch language proficiency. More importantly, as the children asked each other the questions, their interaction increased and the power asymmetry with the researchers decreased. The visibility of the themes stimulated the children’s participation and concentration during the conversation.

The focus groups were held in Dutch by the first author and an Arabic-speaking Syrian female research assistant was present to translate when necessary.^7^ Most children were capable of understanding the questions in Dutch, but many could express themselves better in their mother tongue. Since the main researcher is a white, Dutch woman—who is also perceived as Dutch by the respondents—the presence of the Syrian research assistant was important for building trust and fostering openness among the children in discussing their experiences. All of the focus groups were discussed and reflected upon afterwards with the assistant, who had fled from Syria to the Netherlands too. She provided relevant cultural contexts to children’s answers and shared her interpretations, which improved the data analysis process.

All focus groups were audio-recorded and transcribed verbatim. The software program Atlas.ti was used in the coding process. A codebook was first developed based on the topics and questions in the focus group. Subsequently, new codes emerged during the coding process and relations between the main themes and subthemes were clarified (Bryman, 2008; Silverman, 2010). During the coding process, concepts relating to boundaries appeared to be most suitable for the theoretical analysis. We therefore analysed whether children discussed groups they are or are not part of, where boundaries are drawn, and if these boundaries were constructed by themselves or by others. To increase the validity of our study, we made a constant comparison between codes, memos, transcripts, preliminary findings and theory. The results were discussed with the Syrian research assistants and an audience of family coaches of Syrian families in Rotterdam to examine the accurateness of our results.

## Results

### Social contacts: children mainly have friends with an Arab background

All children who participated in this study said they have friends in the Netherlands, and most of these friendships had been formed at school. Earlier studies (Beirens et al., 2007; Kia-Keating & Ellis, 2007; Selimos, 2017) also show that school is an important place to meet other children and make friends. Children explained that most of their friends have an Arab background; this means that they either also come from Syria or have a background in another Arabic-speaking or Islamic country. Based on the children’s stories, three explanations for this composition of friends can be distinguished. These explanations show which symbolic boundaries are drawn and how social boundaries—in this case having relations of friendship—are related.

First, children mainly had Arab friends because it is difficult to encounter “Dutch” children^8^; they experienced a lack of opportunity to meet them. This can be explained by the fact that all newly-arrived children spend their first period of school—one or two years—in transition classes in order to learn the Dutch language before they integrate into regular classes. Although these transition classes are often located in the same building as the regular classes, the children are mostly surrounded by other newly-arrived children and are relatively segregated from regular pupils (Crul et al., 2016). As a girl explained, *“I don’t have Dutch friends, because my class is only for the language”* (girl, 17 years, fg1). In other words, the education system’s separate transition classes impose a symbolic boundary between “newly-arrived” and “non-newly-arrived” children that affects which friendships can be made. The few children who indicated that they attend activities outside school, such as sport clubs or music lessons, explained that they had made some “Dutch” friends there.

Whereas the symbolic boundary imposed by the educational system provides the first explanation of why the children had a particular composition of friendship groups, the second and third explanations revealed personal preferences. In half of the focus groups, children discussed how it is often easier to be friends with other children with an Arab background because they can express themselves better in their own language. One child told us, “*You can tell her [Syrian friend] more. Because maybe your Dutch is not good enough to say what you want to say [to Dutch friends]*” (girl, 17 years, fg9). Another girl answered, “*It is not different, but with the language and so on, it is easier with Syrians*” (girl, 14 years, fg1). The importance of language was also stressed by boys. Three younger children (aged 10 and 11) stated that language is not important at all, perhaps because interacting with peers at their age requires less advanced language skills.

Alongside linguistic factors, children revealed that cultural factors also determined the composition of their friendship groups. This third explanation was mentioned in two focus groups with older children (14+ years old), where the boys emphasized that they prefer friends with an Arab background because they understand them better. These boys draw a clear symbolic boundary—Arab versus non-Arab—that is linked to the social boundary of being friends with the other person. The boys explained that their Arab friends think like them, have the same ideas, and have a similar sense of humour:

***R5:*** *I have many friends at school […] Only Arab, that’s better for me […] All that come from the Netherlands, they don’t understand me. I don’t know why. But I think, they have another head. Arab thinking …*

***R4:*** *Different opinion.*

***R5:*** *Different opinion.*

***R3:*** *Arab people understand each other*

*(boys, 15–16 years, fg3)*

***R3:*** *So, for example, sometimes I tell a joke to R4 and he starts to laugh. And if I tell exactly the same joke to a Dutch, or to someone who is not from Syria or has the Arab culture, then this persons says: ’Eh … what’s that? [what do you mean?]’*

*(boy, 15 years, fg10)*

When asked to clarify the differences between cultures—as the children called it—the boys found it hard to explain what the differences consist of:

***R3:*** *The differences are … I don’t know what the differences are, actually. But, they just don’t understand us. I don’t know how, how I can explain. I don’t know myself, you know.*

***R4:*** *The feelings, these are different. If I talk to him [R3], our feelings are the same. But with Dutch people, I feel something different.*

*(boys, 15–16 years, fg10)*

This quotation shows that the differences in culture are intuitively present, but difficult to elaborate upon. In discussing whether these cultural differences could be bridged—and thus whether the symbolic boundary between Arab versus non-Arab could be crossed, shifted or blurred—the boys did not agree. One of them articulated that he had hardly met any “Dutch” children because of the transition class, but he expected that more contact with “Dutch” children would lead to a better understanding of one another and could consequently lead to friendship. Other boys did not think this would make a difference. The girls in one of the focus groups did not agree with the idea that Arab friends understand each other better. Instead they stressed similarity in personal values to be more important to friendship than one’s country of origin or ethnic background: “*Sometimes Dutch people understand me better than Arab people, it depends on the person*” (girl, 17 years, fg3). These girls thus draw another symbolic boundary that is based on similarities in values.

Whereas the next paragraph will show that children are also involuntarily subjected to symbolic boundary-drawing by others, the above analysis shows that children actively draw symbolic boundaries themselves when elaborating about their friends. It is important to note that it is not always evident or clear-cut who the children perceive as Dutch and who is perceived as Arab. They often use the category “Arab” to refer to Arabic-speaking children; however, some grouped Turkish or Moroccan children under the same label, as they are also part of “Arab culture”. Who is perceived as Dutch in the highly diverse city of Rotterdam is an even more complicated question. Second or third-generation “migrant” children or “black” children seem to be labelled as foreigners, rather than Dutch, as discussed in two focus groups with older children (15+ years). These notions of “Dutchness” illustrate that symbolic boundaries are constructed, not straightforward, and correspond to the social boundary of who the children prefer to be friends with.

It can be concluded that all of the participating children have friends, and that school is an important context for meeting them. The educational system of transition classes influences the peers they most easily encountered and some children’s preferences also affected their network of friends. Friendships with children from an Arab background can be seen as bonding relationships—ties with children of their own ethnic group—which “are important for a sense of belonging, for learning from others ‘like them’ about getting a feel for the game in the new country” (Correa-Velez et al., 2010, p. 1406). However, bridging relations with the broader host community is important for feelings of belonging towards their new country and are often seen as being important for their successful integration (Beirens et al., 2007). More specifically, bridging relations can connect children to social and economic resources that provide greater opportunities for their education, employment and language development (Correa-Velez et al., 2010). Actually, the majority of children preferred to have more contact with “Dutch” people. Children mentioned this longing in the focus groups, and survey research on Syrian youth (15–21 years) in Rotterdam showed that almost 90% of them would like to have more contact (Van der Ent, 2019).

### Feeling welcome and experiences of exclusion in the Netherlands

Besides peer relations, acceptance in the broader social environment—Dutch or Rotterdam society—is important for how newly-arrived children fare in their new country. Almost all of the children asserted that they feel welcome in the Netherlands, and described Dutch people as being very friendly and helpful. Only two younger boys (9 and 12 years old) did not feel welcome. Children specifically mentioned their kind neighbours, teachers at school and people in the street who smile at them and greet them, even though they do not know each other personally. Moreover, children encountered people who are interested in them:

*Yes, I think I am welcome in the Netherlands. Because people always ask: ‘What’s your dream? Do you have any hobbies? Can we help you with anything?’ (girl, 16 years, fg3)*

Although almost all of the children said that they feel welcome in the Netherlands, more nuanced answers were given by children between the ages of 13 to 17; around one-third of the children who participated in this study indicated that they felt both welcome and not welcome at the same time. Indeed, alongside their positive experiences, they had encountered certain situations in which they felt excluded or viewed as the “other”. Both boys and girls mentioned not being accepted because they are an Arab, Muslim, Syrian, refugee or foreigner. They experienced a symbolic boundary being drawn—they are “the other”—and in many cases, they had been treated differently, which shows the corresponding social boundary. Exclusion is a topic that evoked heavy emotional responses among the children: they raised their voices, came up with longer stories and reacted more intensely to one another. This indicates that exclusion appears to be a topic that matters to them.

In the analysis of the exclusionary practices encountered by children, three types of exclusion can be distinguished. In all three types, children are seen as “the other” (symbolic boundary), resulting in different types of treatment (social boundary). Firstly, children talked about examples of being *the other that is not wanted in the Netherlands*. They encountered people who stared at them in an unfriendly way, scolded them or simply said that they should not be here, but should go back to their own country. The following quotation illustrates these types of encounters, in which people explicitly told the children that their reasons for fleeing are mistrusted and their presence is undesirable:

***R3:*** *Many Dutch people say: you’re welcome in the Netherlands. They are kind. But some are not, they say: “You’re not Dutch, you come from a foreign country. We don’t like you”. That’s my experience.*

***R4:*** *Yes, ’You have your own country’*

***R3:*** *Yes, “Why do you come here?”*

*(girls and boys, 14 years old, fg2)*

As the following quotation shows, being unwanted in the Netherlands can also be more complex. This boy feels only temporarily welcome, as although people are nice to him, he has also been asked several times about his intentions to return to his home country. The Netherlands is not his country of birth, and the boy explained that people have made it clear to him that he does not belong here. Although these questions are not meant to exclude or hurt him per se, he interprets them as a sign that his future should definitely not lie here:

*Yes, and I think … We feel welcome […], but only for a short time. Because they always ask: ‘Do you go back to your country? Don’t forget your country. Don’t forget it, that’s better, the Netherlands is not your country’. Yes, they always ask. (boy, 15 years, fg3)*

Secondly, children mentioned that they are *the other who may not be part of a* *group*. Children gave examples of peers who laughed at them when they made a language mistake and consequently emphasize that they are an outsider. Two girls explained that children at school sometimes ignore them or react differently to their presence because they wear a headscarf. The youngest child who talked about exclusionary practices was 10 years old and was excluded from playing in the street by other kids:

*Yes, if I see boys are playing and I want to join, they say: ‘No you cannot join. Because you’re from Syria, you’re not from the same, you’re not from my country’. (boy, 10 years, fg7)*

Thirdly, groups of older children (15+ years) in particular mentioned that they are sometimes *the other who is not believed or who is unjustly blamed for problems*. In their examples, they showed how they feel stereotyped as Syrian, Arab or refugee. The children noted that they are evaluated as a member of a group with a negative reputation, instead of being seen and evaluated as an individual (Elias & Scotson, 1965; Tajfel, 1982). For example, when neighbours had noise complaints, they would often immediately and unjustly blame the Syrian family, especially the children. In two focus groups, boys and girls mentioned that they are often not believed in a school setting. The girls presented a situation where they were ill and needed to hand in a letter from the doctor to confirm their illness. This is a common practice at Dutch schools, but the girls interpreted this request for a letter as a signal of suspicion and thought that they had to submit the letter because they are Arab and/or Syrian:

***R3:*** *But the teachers don’t believe us!*

***BvdE:*** *Why don’t they believe it?*

***R4:*** *Their thoughts: “All Arab people lie”*

***R3:*** *Yes, they think all Syrian people want to lie, yes, they lie. Yes.*

*(girls, 16–17 years, fg4)*

Older boys (fg3) emphasized that they are unjustly blamed for causing problems in a school setting. For example, if a Syrian child was pushed by another child and he pushed back, often the Syrian child would be blamed because the teacher said he had only seen the Syrian child pushing. The boys in the focus group thought that it was impossible that the teacher had not seen the other child initiating the conflict. Consequently, they felt unjustly singled out as the culprit of the conflict. Two boys (15 and 16 years, fg10) who had lived in the Netherlands for more than 4 years, mentioned that Geert Wilders—the leader of the right-wing political party *PVV—*unjustly blames refugees for problems in the Netherlands. Whereas all other examples of exclusion are encounters on the micro-level, only these two boys mentioned the negative representation of refugees and Muslims in political debates and explained that it influences their feelings of belonging. It is likely that these boys have been more exposed to Dutch media or (political) discourse than the other children in the focus groups, due to their age and time spent living in the Netherlands.

All of the above examples of exclusion involve situations whereby the children were involuntarily subjected to symbolic boundary drawing by others. But the children also participated in boundary work. When talking about their experiences of exclusion, some older boys and girls (15+ years) stressed they are not excluded by the “Dutch”, but only by “foreign” or “black” children. These people may have been born in the Netherlands, but are still “foreign” in the eyes of the children in our study. The following quote shows the symbolic boundary constructed between the perpetrators of exclusion and “Dutch people”, and subsequently how the girls argue that the “foreigners” erroneously claim that the Netherlands is their country:

***R2:*** *Yes, they are Surinamese or Moroccan or Turkish, and they are racists to us, because we are foreigners. But they are foreigners as well.*

***R4:*** *Yes, yes, indeed.*

***R3:*** *Dutch people don’t do like that, they help us. But Turkish people or Moroccan people, they live here for a long time. And then they say: “No, no need [for you to live here]”*

***R2:*** *They thought: the Netherlands is for them.*

***R3:*** *They think they are Dutch people. But no, it is not true. They are foreigners as well.*

*(girls, 16–17 years, fg4)*

To conclude, this section has shown that Syrian-born children predominantly feel welcome in the Netherlands and appreciate its friendly, helpful citizens. At the same time, a significant portion of (mainly older) boys and girls had experienced exclusionary practices, and different types of exclusion can be distinguished. Exclusion fuelled many emotions in the focus groups, which stressed that exclusion is a topic that is relevant to them and affects them emotionally. The analysis showed that children feel subjected to different symbolic boundaries—e.g., Arab, Syrian, refugee, foreigner—that can all lead to the social boundary of different or disadvantaged treatment.

## Discussion and conclusion

In this article, we have explored how newly-arrived children with a refugee background experience living in the Netherlands. Focus groups with 46 Syrian-born children provided insights into their own perspective on their lives in Rotterdam and showed both the predicaments and positive elements in their lives. The first question concerned how these children evaluate their social contacts with peers in the Netherlands. All of the children talked about having positive peer relations. A symbolic boundary was drawn by most children when they explained that their friends mainly come from an Arab background due to the fact that they have greater opportunities to meet each other, while some children expressed a preference for such friendships due to linguistic and cultural similarities. Most of the children wished to have more contact with their “Dutch” peers. The second research question concerned the children’s experiences within the broader social environment in the Netherlands. Overall, they expressed that they feel welcome, and evaluated people as being friendly and helpful. Nevertheless, different forms of exclusion (a social boundary) were discussed, and these feelings evoked strong emotions and frustrations. Exclusion is clearly a topic that matters to these children, especially those who are slightly older, and can be based on different symbolic boundaries, such as being a refugee, Arab, Muslim or Syrian.

This study shows some of the predicaments in the lives of children with a refugee background. The social boundary of exclusion and the different forms in which it is expressed—being the other who is not wanted, who is not part of the group, or who is not believed and unjustly blamed for problems—is a particularly important post-flight stressor that can negatively affect their well-being, mental health and integration in the Netherlands (Correa-Velez et al., 2010; Fantino & Colak, 2001; Van Schie & Van den Muijsenbergh, 2017). Children’s stories of exclusion need to be taken seriously as a negative social climate can have serious consequences. Although the children were positive about their social contacts with mostly Arab friends at school, it should be noted that more bridging connections can be helpful to improve their Dutch language skills and connect them to social and economic resources, which would provide them with opportunities for the future (Beirens et al., 2007; Correa-Velez et al., 2010). Besides the predicament and vulnerability of newly-arrived children, this study also reveals an important, more optimistic story. The children predominantly expressed that they feel welcome in the Netherlands, encounter many helpful people who are interested in them, and had made new friends who act as a source of social support. Overall, their perspective on living in the Netherlands is positive, and this side of the coin also requires attention.

Theoretically, this study contributes to the boundary perspective in several ways. We showed that symbolic boundaries can have different origins. They can be imposed by a social structure, such as the educational system of transition classes. However, they can also be the result of bottom-up processes, for example, when children have personal preferences for making friends. Moreover, this study highlights that the children draw symbolic boundaries themselves, making distinctions between Arab and non-Arab or Dutch and non-Dutch. However, they are also involuntarily subjected to boundary work, for example, when they are categorized as Syrians and refugees. It became clear that a similar symbolic boundary, such as Arab versus non-Arab, can be used by children and experienced positively—such as in the case of making friends—but can also be experienced negatively when this category is used by others as grounds for social exclusion or treatment putting them at a disadvantage. Symbolic boundaries can thus have diverse consequences for various corresponding social boundaries.

Even if this study and the method of focus groups used offers relevant insights into the perspectives of newly-arrived children, it has its limitations and could be critiqued on several points. First, when exploring children’s perspectives, a power asymmetry between participants and researchers always poses a challenge. By using a board game as a research tool and letting children ask each other questions, we tried to minimize this imbalance and strengthen the children’s ability to describe their experiences in their own way. Second, although we did our utmost best to make the focus groups a safe and comfortable setting for all children, group dynamics or hierarchies between children can have an influence on the stories they share. The presence of a white, Dutch woman (as the main researcher) and a Syrian-born woman (as research assistant) could also have an effect. We deliberately chose this mixed-origin team of researchers—especially as it can be difficult to have only a Dutch researcher when discussing feeling welcome in the Netherlands—and we have the impression that it helped children to share both positive and negative experiences openly. Third, awareness of the context of our findings is important. In this case study, the focus was on Syrian-born children in Rotterdam who are living with their families. This specific historical and societal context must be taken into account. Although all children share their country of birth, it is important to be aware that they can be heterogeneous on other characteristics (e.g., flight experiences, religion, their family’s socio-economic status). At the same time, it is likely that other newly-arrived children have partially similar experiences as Syrian-born children because they share being newly arrived. This implies a broader relevance of the findings in our study.

The findings of this study give rise to new questions. Particularly, the concept of symbolic boundary work requires more attention. How do newly-arrived children in a diverse city such as Rotterdam use the labels “Dutch” and “non-Dutch”, and what are the implications? How do children cope with the symbolic boundaries they are subjected to (e.g., being seen as a refugee, Arab, foreigner) and with the corresponding social boundaries (disadvantaged treatment or exclusion)? Regarding the domain of social contacts, it would be interesting to study how the composition of their friendship groups develops after they leave the transition classes. Additionally, whereas feeling welcome is an important starting point for newly-arrived people, it is not the same as being fully included or accepted in society. It is relevant to see whether feeling welcome develops into (full) inclusion over the coming years. Surveys and longitudinal qualitative research will be conducted within the larger research project to elaborate on these research questions.

Methodologically, this study contributes an innovative research tool developed by the first author, which is connected to children’s life worlds and related to their capacities. Using a board game with question cards made the focus groups fun and interactive, increased children’s participation and diminished the power asymmetry between the children and researchers. The research tool made it possible for children to share their perspectives regarding their peer relations and the broader social climate. These insights into how they perceive their own lives form the main empirical contribution of this study. Imagine once more that you are a Syrian-born child forced to flee the war, who is now living in the Netherlands. If the stories of the children in this study have helped you to see the world a bit more from their point of view, then the aim of this article has been accomplished.

## Notes

1. A person with a refugee background is “someone who has been forced to flee his or her country because of persecution, war or violence” (www.unrefugees.org, 2019). All respondents in this research are acknowledged as refugees and have a temporary residence permit in the Netherlands.

2. This interview was meant to be a focus group, but only one child attended instead of the five invited children. The same method with the same questions was used to talk about the child’s experiences.

3. In the analysis, we looked closely at possible differences between the diverse groups in terms of gender, age, group composition, the research setting, and whether the child was participating in the SNTR programme. Any differences that we found are mentioned and described in the paper.

4. Five focus groups were conducted either at a summer school for Syrian children, or in transition classes at a secondary school. In these cases, we had access to a list of the children’s names. Their parents were informed by letter and asked for consent. Six focus groups were conducted at a language school for parents or a refugee organization. For these focus groups, we asked parents to bring their (mostly younger) children to class or to appointments with the organization, so their children could participate in the study.

5. This study was approved by the ethical committee of the department of Public Administration and Sociology at the Erasmus University Rotterdam.

6. The other themes—a treasure chest for individual answers, the situation at home, their dreams and expectations for their future, and giving advice to policy makers and Syrian children to improve the situation of newly-arrived children—are beyond the scope of this paper and will be elaborated upon in future papers.

7. Arabic answers were immediately translated into Dutch and quotations were translated into English for this article.

8. “Dutch” is written between inverted commas because the children had complex notions about who is actually Dutch. We reflect on this at the end of this paragraph. When “Dutch” is written in this paper, it refers to how Syrian children in the focus groups used the category.
